# Seventh Day Syndrome Revisited: Early Recognition of the Clinical Syndrome and an Evolving Understanding of Its Etiology

**DOI:** 10.3389/frtra.2022.913584

**Published:** 2022-06-28

**Authors:** James M. Halle-Smith, Lewis A. Hall, Angus Hann, Hermien Hartog, M. Thamara P. R. Perera, Desley A. H. Neil

**Affiliations:** ^1^Liver Unit, Queen Elizabeth Hospital, Birmingham, United Kingdom; ^2^College of Medical and Dental Sciences, University of Birmingham, Birmingham, United Kingdom; ^3^Department of Cellular Pathology, Queen Elizabeth Hospital Birmingham, Birmingham, United Kingdom; ^4^Institute of Immunology and Immunotherapy, University of Birmingham, Birmingham, United Kingdom

**Keywords:** liver transplant, seventh day syndrome, antibody mediated allograft rejection, liver transplantation, rejection

## Abstract

**Background:**

Unexplained acute failure of an initially functioning liver graft early post-transplant has been described as Seventh-Day Syndrome (7DS). The aims of this study were to describe the clinical syndrome in detail based on an institutional case series and literature review.

**Methods:**

A retrospective review of adult patients that underwent deceased donor liver transplantation at our institution between January 2010 and 2020 was performed to identify patients that developed 7DS. Relevant clinical variables were obtained from medical records. Existing cases in the literature were identified by a systematic literature search according to PRISMA guidelines. Pooled analysis was used to describe the incidence, retransplantation, and mortality rate. Histological findings from institutional and published literature cases were collected and appraised.

**Results:**

Six of 1,907 liver transplantations at our institution (0.3%) developed 7DS. Seven case series, describing 42 patients with 7DS, and two single case reports were identified from literature review. Pooled incidence of 7DS was low (2.1%, 95%CI: 0.7–3.9%) and associated with high mortality (74.8%, 95%CI: 49.2–94.6%). Retransplantation was performed in 23/42 (55%) patients and 4/23 (17%) survived. Review of histology showed frequent intrahepatic thrombi and arteritis. Rejection, with features of potential antibody mediated rejection, often preceded or accompanied progressive zonal coagulative necrosis and hemorrhage.

**Conclusions:**

7DS is a rare clinical syndrome after liver transplantation and associated with high mortality. Antibody-mediated rejection, as suggested in early reports, is likely to be involved in the pathogenesis. Early recognition would allow rapid clinical diagnostics and expedited decisions, such as treatment of AMR if diagnosed or early retransplantation.

## Introduction

Acute graft failure early following liver transplant is a challenging clinical scenario, requiring prompt diagnosis and the appropriate management. The spontaneous failure of a liver graft with previously improving function and patent vasculature, typically occurring within the initial 14 post-operative days, is a rare but recognized entity. This clinical syndrome was labeled as the Seventh Day Syndrome (7DS) by Memom et al. (1), and several earlier case reports fit into the same description of 7DS (2, 3). Graft failure that necessitates retransplantation has an incidence of 10% following liver transplantation, with approximately 40% of retransplants being performed early in the first month on an urgent basis (4). The causes of graft loss in the first week after liver transplantation are predominantly due to primary non-function (PNF) and hepatic artery thrombosis (4). Despite identification of this syndrome in both living and deceased donor liver transplantation settings, the pathophysiology remains poorly understood (5–8).

Early super-urgent liver re-transplantation is associated with a mortality between 25 and 35% (4, 9). In addition, there is a further mortality related to the lack of a suitable donor liver becoming available in an acceptable timeframe. PNF is the leading cause of graft loss in the first 7 days following liver transplantation, attributed to use of extended criteria donors and the accompanying ischemia-reperfusion injury (IRI) process which negatively affects many aspects of cellular function (10). Several definitions for PNF exist in the literature but they all have the common theme of graft failure, with signs of dysfunction almost immediately evident following transplantation and requiring retransplantation or leading to death, without another identifiable cause (11). Grafts that experience PNF fail immediately after transplantation without evidence of normal or improving function. The distinguishing feature of 7DS is that graft failure occurs following an initial period of adequate function. Significant progress has been made over the years regarding the prevention of PNF and its incidence is consequentially decreasing (12). However, knowledge regarding the risk factors, pathogenesis and effective graft salvaging treatment options for 7DS is lacking at present.

Several terms have been used to describe sudden and unexplained graft failure after liver transplantation in the absence of vascular issues, infection or immune infiltrate consistent with T-Cell mediated rejection (TCMR) (13). In addition to TCMR and since the original description of 7DS, antibody mediated rejection (AMR) has gained increased recognition over recent years (1, 14, 15). Interestingly, two early publications (3, 16) allude to a link of 7DS with rejection, a conclusion also reached later by McCaughan et al. (17). Therefore, our aim was to describe the cases of 7DS from our institution, review the available literature and compare all available histological data with the recent descriptions of AMR. Our hypothesis was that AMR is implicated in the pathogenesis of 7DS. Understanding the pathogenesis would be of benefit to transplant clinicians as it will provide direction for the most appropriate management strategies, which may provide alternative algorithms to explore potential treatment options, minimize graft loss and patient death following presentation of this disastrous syndrome.

## Materials and Methods

### Institutional Case Series

A retrospective review of adult patients that underwent deceased donor liver transplantation at our institution between January 2010 and 2020 was performed to identify patients that developed 7DS. Relevant clinical variables such as observations, blood test and imaging results were accessed through electronic medical records. Histopathology data were accessed through the histopathology laboratory records and slides were reviewed contemporaneously where available. Slides from biopsies, but not the failed allografts of the original Birmingham series describing massive haemorrhagic necrosis, were also still available for review (3).

### Literature Search

**Figure 1:**
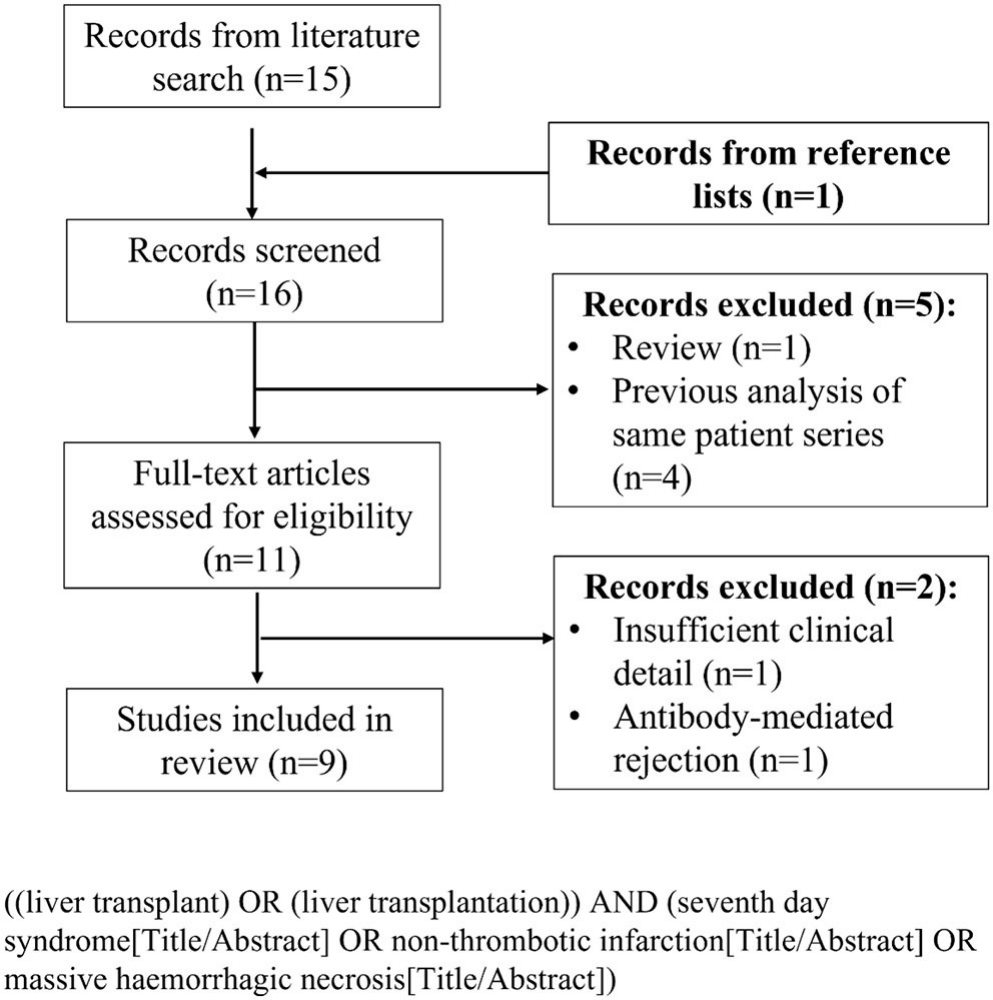
PRISMA flow diagram of included studies and search term.

A formal literature search was conducted according to the PRISMA guidelines (18). The PubMed and EMBASE databases were searched in July 2021 to identify studies reporting cases of 7DS following liver transplantation. The initial search was restricted to title and abstract. Titles, abstracts and subsequently full-text articles were screened according to pre-defined inclusion and exclusion criteria. Studies were excluded if they were review articles, did not report any cases of 7DS or lacked sufficient clinical descriptions of 7DS cases. Studies that reported graft failure because of AMR following liver transplantation, based on accepted criteria (15), were excluded. Reference lists of included studies were cross-checked to identify any additional relevant studies. If multiple studies were published from the same institution within a similar study time period only the most recent was included, for example in the case of two studies from Lan et al. (5) and Zhongwei et al. (7). The search term and PRISMA flow diagram of included studies are displayed in Figure 1.

### Data Collection From Published Literature

The full text manuscripts of the included studies were obtained and independently reviewed by three authors (JHS, LH, AH) and relevant variables collected with a standard data collection tool. Any differences between authors in data review and collection were resolved by consensus. Donor and graft details, recipient demographics, clinical presentation, investigation results, pathology findings, treatment administered and outcomes of patients with 7DS in the included studies were collected and reviewed. The day of syndrome onset was defined as the post-operative day the patient developed significant elevation in their liver function tests (LFTs). A pooled analysis was performed to determine the incidence and outcome of 7DS.

## Results

### Institutional Case Series

**Figure 2:**
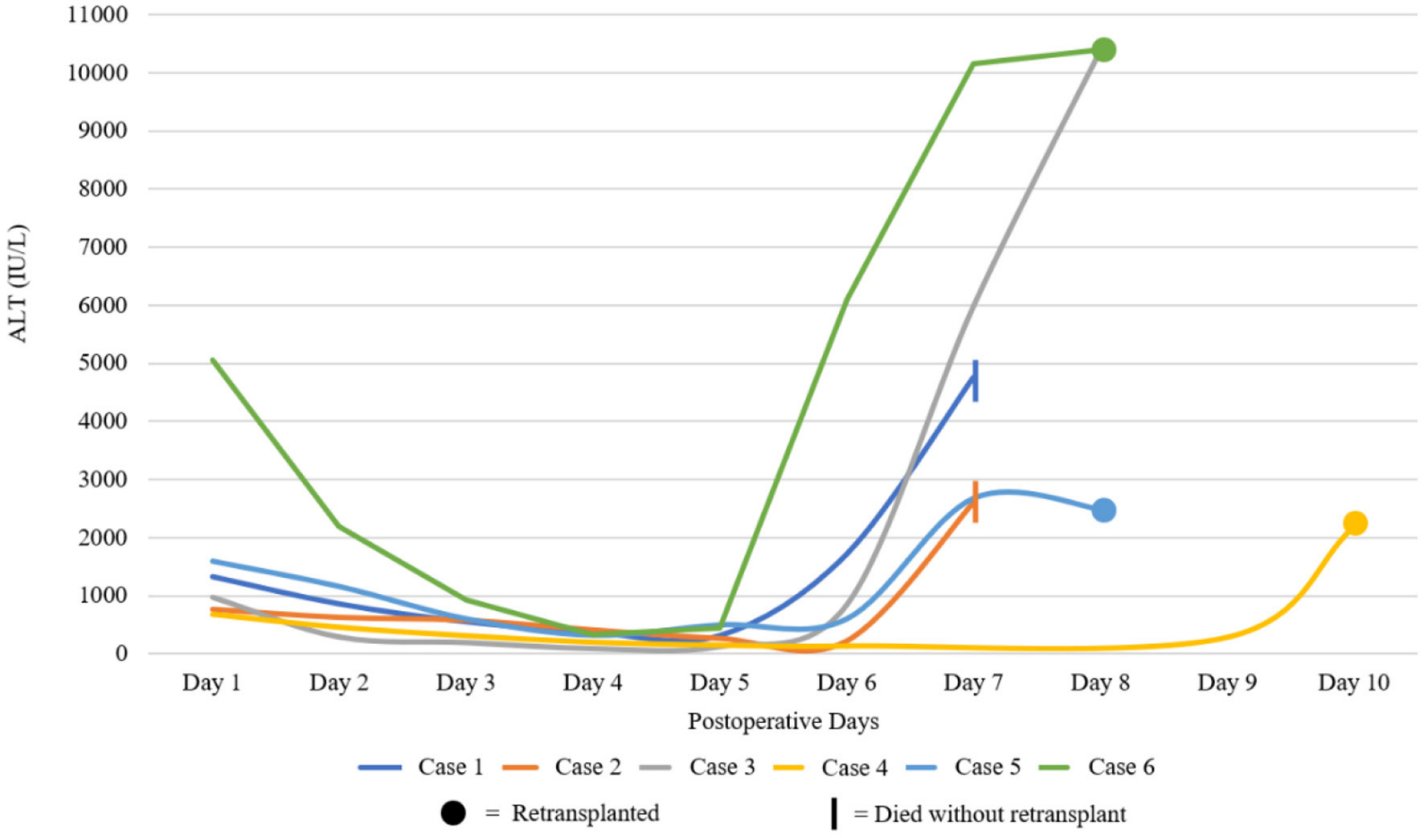
Graphical demonstration of post-operative liver function. Alanine aminotransferase (ALT) for each case during the initial post-operative period. Graph line ends at either death or retransplantation. AST and not ALT depicted for Case 3 and Case 6.

Between January 2010 and April 2020, 1907 deceased donor liver transplants were performed. During this period, 6 patients (6/1907, 0.3%) experienced 7DS and either died or required retransplantation (Table 1A). The recipient age ranged from 21 to 70 years old and the majority of grafts (5/6, 83%) were from deceased brain death (DBD) donors. There was no assessment of pretransplant sensitization in the form of tissue cross matching or anti-HLA antibodies for any of these patients. The immunosuppression regime for all patients was initiated in the post-operative period and comprised Tacrolimus, an antimetabolite (Azathioprine or Mycophenoilate Mofetil) and corticosteroids. Following a decline in transaminases in the initial post-operative period and stable clinical course, all patients displayed a significant and abrupt elevation of their liver enzymes. In all cases in which ALT was measured, there was an acute rise to >1,000 IU/L. A solitary patient (Case 3) did not have post-operative ALT levels performed, however the aspartate aminotransferase (AST) levels reached a maximum of 10,390 IU/L. The onset of the syndrome occurred between post-operative day 5 and 10 and rapidly progressed (Table 1A; Figure 2) in all cases. Vascular complications were excluded in all patients by both duplex ultrasound and CT. Five of the 6 (83%) had a fever (≥38°C) at the onset of clinical deterioration. All patients received high-dose corticosteroids and were managed in the intensive care unit with supportive care. Retransplantation was performed in 4/6 (67%), one patient (1/6, 17%) was waitlisted urgently for retransplantation but did not receive a graft within a suitable time period and died. Retransplantation was deemed futile in another patient (1/6, 17%) due to severity of illness. In the group that underwent retransplantation, this was performed between 24 and 72 h after deterioration (Table 1A).

**Table 1A:** Clinical characteristics of seventh-day syndrome cases at the Liver Unit, Birmingham.

**Case number**	**Donor type**	**Age**	**Gender**	**Transplant indication**	**Day of presentation^†^**	**Day of presentation**		**Peak ALT**	**Fever**	**Day of biopsy and histology**	**Postoperative day retransplanted**	**Mortality** **<90 days**
						**Base excess (mmol/L)**	**Lactate (mmol/L)**					
1	DBD	31	F	Seronegative hepatitis	5	−14.2	14.4	3,342	Yes	Not applicable	Not applicable	Y
2	DCD	70	M	NASH with HCC	7	−12.7	17.9	2,599	Yes	Not applicable	Not applicable	Y
3	DBD	56	F	Hepatitis C cirrhosis	6	−1.3	6	10,390^‡^	No	Day 7. Non-inflamed portal tracts and zone 3 coagulative necrosis	Day 9	N
4	DBD	60	M	HCC	10	−7.6	10.1	2,195	Yes	Not applicable	Day 11	N
5	DBD	36	M	Budd Chiari Syndrome^§^	7	−0.7	11	2,673	Yes	Not applicable	Day 10	N
6	DBD	18	F	Seronegative hepatitis	6	−8.2	10.4	3,543	Yes	Not applicable	Day 9	N

† *As evidenced by onset of ALT elevation*.

‡ *AST reported as ALT result not available*.

§ *Patient had a known JAK2 mutation and received therapeutic anticoagulation post operatively*.

*DBD, deceased brain death; DCD, deceased circulatory death; NASH, non-alcoholic steatohepatitis; ALT, alanine aminotransferase*.

**Figure 3:**
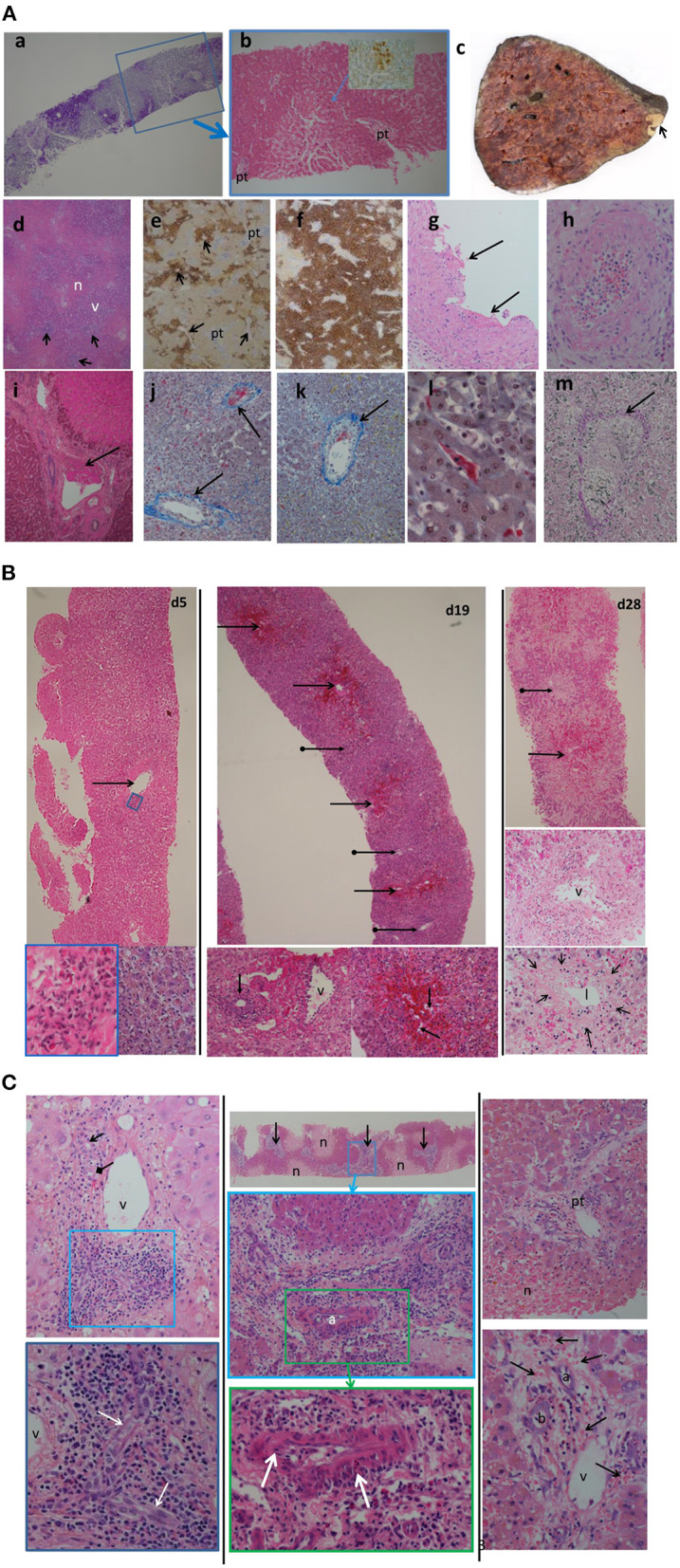
**(A)** Histological appearances of specimens from patients with seventh-day syndrome at our institution. Images from our recent cases series [case 3 (a,b,i); case 4 (c–h,k–m)]. (a) Periodic acid Schiff (PAS) stain. (b) Haematoxylin and Eosin (H&E) stain of day 7 post-transplant biopsy of case 3. There is severe glycogen depletion of the liver with residual glycogen seen as the deep pink/purple color. The area outlined by the blue box is show in (b). The hepatocytes surrounding the portal tracts (pt) are a darker pink than the hepatocytes between. The arrow points to the area seen in the inset with a few necrotic hepatocytes detected by C4d staining (brown). (c) The macroscopic appearance of failed allograft in case 4. A small peripheral infarct is seen as the pale area (arrow) the rest of the liver has a mottled deep red congested haemorrhagic appearance. No macroscopic thrombi were identified. (d) (H&E) and (e,f) (C4d) show the extent of hepatocyte necrosis: the necrotic (n) areas are paler pink with a zonal (2, 3) distribution, inflammation of portal tracts is apparent as darker purple staining (arrows), the periportal zone 1 viable (v) hepatocytes staining a purple compared to the necrotic (n) hepatocytes. The extent of the necrosis varied throughout the liver with predominantly necrotic hepatocytes (brown staining) found around hepatic veins (arrow). The portal tracts (pt) stand out as slightly blue. In (f), there is more extensive panacinar necrosis in most areas. Fibrin thrombi are seen in hepatic arteries (G), portal veins (i), hepatic veins (j,k), and sinusoids (l). (g,i) H&E stain thrombi (arrows). Intimal inflammation is seen under the mural thrombi in (g). The artery in (h) (H&E) shows fibrinoid necrosis and arteritis (arrow). (j–l) Martius Scarlet Blue (MSB) stain in which fibrin is red and collagen is blue. Inflammatory hepatic vein lesions with intimal oedema (inflammatory non-occlusive veno-occlusive lesions) are seen in (j,k), with the wall of the vein outlined by blue collagen (arrow). (l) Sinusoidal thrombus (red) is present in areas of viable hepatocytes as evidenced by the hepatocyte nuclei. (m) Elastic haematoxylin van Giesson (EHVG) stains collagen pink (arrow) outlining a hepatic vein which is occluded (venocclusive lesion) in an area of haemorrhagic necrosis (red bloods cells green). **(B)** Images of sequential biopsies from case 4 in the massive haemorrhagic necrosis paper (Hubshcer et al.) from our institution showing increase zonal necrosis over time. Left column day 5 post transplant, central column day19 and right column day 28. In the day 5 biopsy there are scattered necrotic eosinophilic hepatocytes and associated mild lobular inflammation (bottom right left column). There is hepatic venous inflammation (arrow) in the top left image with a higher power of the area within the blue rectangle in the bottom left image. Eosinophils are admixed with lymphocytes. By day 19 (central column) there is zone 3 haemorrhagic necrosis (arrows) with mild inflammation of the portal tracts (arrows with globular end), shown at higher power in the bottom left image of the middle column. There is evidence of acute T cell mediated rejection with bile duct (arrow) inflammation and portal venous (v) inflammation. In the lower right image of the central column the zone 3 hemorrhage (red) is within hepacyte plates in some areas as evidenced by the empty sinusoids In the right column there is more extensive zone 2 and 3 haemorrhagic necrosis (arrow). Portal tract (arrow with globular end) with the portal tract. The central image of the right column shows a portal tract with the portal vein (v). A bile duct is not identified. The lower image of the right column shows a hepatic vein with the fibrous wall outlined by the arrows and the lumen (l) narrowed due to the intimal expansion (*) by oedema, red cell extravascation and a few mononuclear cells. **(C)** Images from 3 cases in the massive haemorrhagic necrosis paper from our institution (Hübscher et al.) showing different features suggesting rejection, in particular AMR. Left column: case 6 from biopsy day 9 post-transplant, 5 days before the regraft. Portal tract with a normal bile duct (arrow) and artery (diamond tip arrow) and portal vein (v). toward one end of the portal tract there is increased inflammatory infiltrate (blue box) which is enlarge in the lower image. Within this area there is a “dysplastic” sick bile duct. Central column: case 5 from a biopsy 3 days post retransplant for “MHN” and 3 days before retransplantation. At the top is a low power image showing pale zone 2 and 3 necrosis (n) with the viable hepatocytes staining dark pink. The portal tracts (arrows) are inflamed. The central image is a higher-poser of an inflamed portal tract (blue box) in the lower image. The there is an arteritis of the hepatic artery (a) shown in more detail in the lower image with arrows pointing to areas of inflammatory cells within the intima and media. Left column: Case 2 from biopsy taken 5 days post-transplant and 2 days before retransplantation. Top image lower power showing an oedematous portal tract (pt) and areas of necrosis (n). Lower image is a higher magnification of the portal tract showing the portal oedema in which there is hemorrhage. The artery (a), bile duct (b) and portal vein (v) are seen.

For these 6 cases the histopathology findings are summarized in Table 1B. No pathological examination was undertaken ante- or post-mortem in the 2 patients that died. Failed allografts were examined in the four cases that underwent retransplantation. One case also had a percutaneous liver biopsy taken on day 7, which was 3 days prior to retransplantation and within 24 h of marked increase in transaminases. Detailed histological analysis of specimens from the present case series is shown in Figure 3A. In the biopsy specimen necrosis was present with only very focal extravasation of red blood cells into the space of Disse/hepatocyte plates. Portal oedema, without portal hemorrhage, was present. Mixed zonal and geographic necrosis was present in all the failed allografts, 3 of which also had extensive hemorrhage. Two of the four (50%) had evidence of TCMR, one of which was mild and the other severe. Venous microthrombi were present in all failed allografts (100%) with sinusoidal thrombi in two (50%) (Figure 3A). In two of the failed allografts, features of nodular regenerative hyperplasia were identified. In one of these, this was not apparent on a post-reperfusion or the day 7 biopsy, there was no previous biopsy in the other. Histological appearances of specimens from the previous case series published at our institution by Hübscher et al. (3) are also shown for comparison in Figures 3B,C. The four patients (4/6, 67%) that were retransplanted, all survived greater than 90 days. The two patients (2/6, 33%) that were not retransplanted died within 48 h of syndrome onset (Table 1A).

**Table 1B:** Histology findings for cases of 7DS at the Liver Unit, Birmingham.

**Case**	**Histology sample**	**TCMR**	**Portal oedema**	**Portal hemorrhage**	**Portal microvasculitis**	**Portal eosinophils**	**Arteritis**	**Ductopenia**	**Eosinophilic venulitis**	**Venous microthrombi**	**Arterial microthrombi**	**Sinusoidal microthrombi**	**V-O lesions**	**Hepatocyte necrosis**	**Hemorrhage**
1	None	–	–	–	–	–	–	–	–	–	–	–	–	–	–
2	None	–	–	–	–	–	–	–	–	–	–	–	–	–	–
3	Bx d7	N	Y	N	N	N	N	N	N	N	N	N	N	Y	N
	FA d10	N	Y	Mild	N	N	N	N	N	Y	N	N	N	Y	Y
4	FA d9	Moderate /severe	Y	Mild	Focal	Y	N	N	N	Y	N	Y	N	Y	Congestion
5	FA d11	Borderline/ mild	Minimal	Mild	Focal	N	N	N	N	Y	N	Maybe	Y	Y	Y
6	FA d9	N	Y	Marked	Focal	N	Y	N	N	Y	N	Y	N	Y	Y
			3/4	4/4	3/4	1/4	1/4	0/4	0/4	4/4	0/4	2/4	1/4	4/4	3/4

*TCMR, T-cell mediated rejection; FA, failed allograft; Bx, biopsy; **–**, no pathology sample*.

### Systematic Review of Published Literature

The systematic literature search returned 15 studies. Review of the reference lists returned one extra study for review. Following abstract review, 11 studies proceeded for full-text review. After full-text review, nine studies were identified as suitable for inclusion in the literature review according to the pre-specified inclusion and exclusion criteria (Table 2) (1, 3, 6–8, 17, 19–21). These consisted of seven case series and two single case reports. All nine included studies were retrospective in nature and within these there were a total of 44 reported cases of 7DS (Table 2). There was heterogeneity amongst the included studies in terms of variables reported.

**Table 2:** Clinical characteristics and outcomes of patients with seventh-day syndrome reported in previously published case series and case reported.

**Author**	**Cases**	**Incidence (%) (cases/transplants)**	**Donor type**	**Female**	**Indication**	**Day of presentation**	**Peak ALT (IU/L)**	**Fever**	**Retransplanted (%)**	**Survived without retransplant (%)**	**Mortality (%) (<90 days)**
Hübscher et al. (3)	6	6.0% (6/100)	DDLT	5 (83%)	PBC = 3. A1AT = 1. Cryptogenic cirrhosis = 2.	Day 3 to 20	AST median increase 7.5 × (Range 3 - 14 × ).	Not reported	4 (67%)	0 (0%)	100%
McCaughan et al. (17)	8	5.2% (8/154)	DDLT	2 (25%)	PSC = 2. ALD = 2. Wilsons = 1. HBV = 1. AIH = 1 Drugs = 1	Mean Day 6	Mean maximum 16,000	8 (100%)	2 (25%)	2 (25%)	75%
Burke et al. (19)	4	2.7% (4/150)	DDLT	2 (50%)	HBV = 1. HCV = 1. ARLD = 1. PSC = 1.	Day 5 to 6	2,500–6,000	4 (100%)	3 (75%)	0 (0%)	50%
Memon et al. (1)	10	1.7% (10/594)	DDLT	4 (40%)	ARLD = 4 PBC = 3 Viral hepatitis = 3	Day 4 to 11	1,400**AST	Not reported	8 (80%)	1 (10%)	30%
Hwang et al. (6)	3	0.5% (3/580)	LDLT	0 (0%)	HBV with HCC = 2. ALF = 1	Day 5 to 6	1,852–4,932	3 (100%)	0 (0%)	0 (0%)	100%
Zhongwei et al. (7)	8	3.3% (8.244)	LDLT	0 (0%)	HCC = 3. ALF = 1. Viral hepatitis = 4.	Day 8 to 12	6,300–10,000 some > 10,000	Not reported	0 (0%)	1 (13%)	88%
Pereira et al. (8)	3	Not reported	DDLT	2 (67%)	PSC = 1. Wilsons = 1. HCV = 1.	Day 7 to 8	7,500–15,000	3 (100%)	2 (67%)	0 (0%)	100%
Matsuura et al. (20)	1	Not reported	DDLT	Female	Chronic rejection	Day 7	3,960	Present	No – Desensitization Therapy	N/A	Alive
Qazi-Arisar et al. (21)	1	Not reported	DDLT	Male	Autoimmune hepatitis	Day 6	5,699	No reported	Retransplant	1 (100)	0%

*ALF, acute liver failure unknown cause; AIH, autoimmune hepatitis; ALT, Alanine aminotransferase; DDLT: deceased donor liver transplantation; LDLT, living donor liver transplantation; ARLD, alcohol related liver disease; PBC, primary biliary cirrhosis; HBV, hepatitis B virus; HCV, hepatitis C virus; HCC, hepatocellular carcinoma; ALF, acute liver failure; PSC, primary sclerosing cholangitis*.

Amongst the seven case series, there were 42 cases of 7DS reported (Table 2), 15 (36%) were female and there were nine different indications for liver transplant (Table 2). Viral hepatitis and the associated complications were the most common indication (13/42, 31%). The post-operative day the patients developed acute graft dysfunction ranged from post-operative day 4 to 20. Fever was described as a common clinical feature in several studies (Table 2) (6, 8). All patients developed a significant elevation of their liver enzymes, with an ALT exceeding 10,000 IU/L in majority of cases (Table 2). In all reported cases, the vasculature was shown to be patent at the time of graft dysfunction. The cross-match result was only reported in 23 cases, with 8 positive (35%). Two of the cases reported received ABO incompatible grafts.

The most frequent reported histological findings on either liver biopsy or explanted liver specimens were extensive hepatocyte necrosis. Inflammatory cell infiltrate was reported in several patients, however this was described as minimal. One patient had evidence of rejection on a liver biopsy taken before repeat liver transplantation, then subsequent histology on the explanted liver revealed massive hepatocyte necrosis (Table 3). Arteritis, microthrombi and veno-occlusive lesions were frequently present when the pathology is described in detail in the reports. In the majority of studies there were few histological findings reported (Table 3).

**Table 3:** Summary of histological findings from previously reported cases of seventh-day syndrome.

**Author and Year**	**Cases**	**ABO-I**	**Positive X-match**	**Anti-HLA antibodies**	**Preceding BPAR**	**Histology sample**	**Arteritis**	**Ductopenia**	**Venous microthrombi**	**Arterial microthrombi**	**V-O lesions**	**Hepatocyte necrosis**	**Hemorrhage**	**IH**
Hübscher et al. (3)	6	1/6	N/R	N/R	3/6	Whole liver: 6	3/6	1/6	2/6	1/6	4/6	6/6	6/6	N/R
McGaughan et al. (17)	8	0/8	3/8	N/R	3/8	Whole liver: 8	2/8	N/R	N/R	4/8	3/8	8/8	8/8	IgG, IgM, fibrin, C3 deposition in 2 cases
Burke et al. (19)	4	0/4	0/4	N/R	4/4	Whole liver: 3; Biopsy:1	N/R	N/R	N/R	N/R	N/R	4/4	4/4	N/R
Memon et al. (1)	10	1/10	4/10	N/R	0/6	N/R	N/R	N/R	N/R	N/R	0/10	N/S	N/S	No evidence of IgG, IgM or C3
Hwang et al. (6)	3	0/3	N/R	N/R	2/3	Biopsy: 3	N/R	N/R	N/R	N/R	N/R	N/R	N/R	N/R
Zhongwei et al. (7)	8	0/8	N/R	N/R	0/8	Biopsy: 4^†^	N/R	N/R	N/R	N/R	N/R	4/4	N/R	Increased expression of Fas around portal tracts
Pereira et al. (8)	3	N/R	N/R	N/R	0/3	Whole liver: 3; Biopsy:1	N/R	N/R	N/R	N/R	N/R	3/3	1/3	N/R
Mastsuura et al. (20)	1	0/1	1/1	0/1	0/1^‡^	Biopsy: 1	N/R	N/R	N/R	N/R	N/R	1/1	1/1	CD3 and CD20 staining or portal area^§^
Qazi-Arisar et al. (21)	1	1/1	N/R	N/R	0/1	Whole liver: 1	N/R	N/R	N/R	N/R	N/R	1/1	1/1	N/R

† *Only 4/8 pts had samples analyzed*.

‡ *Indeterminate for AMR*.

§ *PRA, positivity against HLA class II high. CD8 staining predominant compared to CD4. C4d staining negative*.

*ABOi, ABO incompatible; HLA, human leucocyte antigen; V-O, veno-occlusive; IH, immunohistochemistry; N/R, not reported; N/S, not specified; BPAR, biopsy proven acute rejection*.

Immunohistochemistry was performed and reported in three of the studies (Table 3). Intense *Fas* ligand binding demonstrated around the portal tracts in one study, whilst evidence of IgG, IgM, complement and fibrin deposition was not consistent.

There were two single case reports of 7DS reported (Table 2). Both cases followed a deceased donor liver transplant (DDLT). The indication in a 19 year-old female was chronic rejection of a previous transplant, and autoimmune hepatitis in a 47 year old male. Both cases presented at a similar time postoperatively, with significantly elevated transaminases (Table 2). Vasculature was patent in both cases at the time of graft dysfunction and histology revealed massive hepatocyte necrosis with minimal immune cell infiltrate. Immunohistochemistry was performed in one case which demonstrated CD3 and CD20 staining of the portal area. One case was successfully treated with desensitization therapy, whilst the other was retransplanted with an ABOi graft. Both cases were alive at the time of publication (Table 2).

### Pooled Incidence and Outcome Rates

A pooled analysis of the existing published cases of 7DS, and the additional series described in this report, demonstrated the incidence to be 2.1% (95%CI: 0.7–3.9%, I^2^ 87.8%). The pooled 90-day mortality as a result of 7DS was 74.8% (95%CI 49.2–94.6%, I^2^ 61.2%). Retransplantation was performed in 23/48 (55%) patients (Table 4).

**Table 4:** Pooled incidence, retransplantation rate and perioperative mortality rate of seventh-day syndrome (excluding individual case reports).

	**Incidence**		**Retransplant rate**		**Mortality** ** <90 days**	
	**Number of cases**	**Total liver transplants**	**Number of cases**	**Total cases of MHN/7DS**	**Number of deaths**	**Total cases of MHN/7DS**
Hübscher et al. (3)	6	100	4	6	6	6
McCaughan et al. (17)	8	154	2	8	6	8
Burke et al. (19)	4	150	3	4	2	4
Memon et al. (1)	10	594	8	10	3	10
Hwang et al. (6)	3	580	0	3	3	3
Zhongwei et al. (7)	8	244	0	8	7	8
Pereira et al. (8)	3	NR	2	3	3	3
Birmingham (2020)	6	1,907	4	6	2	6
Meta-analysis rate (95%CI)	2.1% (0.7–3.9%)		43.6% (15.9–73.2%)		74.8% (49.2–94.6%)	
I^2^	87.8%		70.5%		61.2%	

*I^2^ represents the percentage of variability due to study heterogeneity*.

## Discussion

Since the original description of 7DS, the number of reported cases in the literature remains very low and little progress has been made in the understanding of the pathophysiological process involved. Due to its extremely rare incidence, 7DS is not mentioned in large cohorts reporting outcomes after liver transplantation, limiting the current evidence of 7DS to case series and case reports of the syndrome. 7DS may be an underreported clinical entity and a meaningful analysis of risk factors, preventative and management strategies remains very challenging. This is further confounded by a lack of detailed information and frequent absence of a tissue diagnosis. However, the present study shows significant progress in the understanding of the pathophysiological process involved, by systematic appraisal of clinical details, histologic evidence and anecdotal reports of successful salvage treatments. The results of our study support previous hypothesis that 7DS may be related to an unidentified immune response and provides further evidence of antibody mediated rejection.

Clinical presentation of cases consistently showed very similar features of acute graft failure with high transaminases on days 4–12, after initial adequate graft function. No particular patient or graft characteristic was clearly associated with 7DS. Interestingly, fever was reported as a frequent prodrome and could be a hallmark feature of 7DS. The 74.8% pooled mortality rate associated with 7DS is very high compared to other causes of early graft failure (22, 23). Two Asian case series, representing 26% of reported cases, had potentially very limited access to urgent retransplantation due to reliance on a living donor liver transplantation program in the absence of deceased donors (24). Survival following retransplantation was higher at our institution (4/4, 100%) and the interval to urgent retransplantation short, compared to the previously published series (7/23, 30%). However, low number of events limit meaningful comparisons. In the initial case series that initially described massive haemorrhagic necrosis by Hübscher et al., there were no survivors despite 4/6 patients being retransplanted within a short period (1–3 days) of illness onset (3). Prompt exclusion of other causes of graft failure such as HAT, and early identification and diagnosis of 7DS may facilitate early relisting or early initiation of potential therapies. Rapid decisions may be the only way to improve chances of survival given the often catastrophic course of the condition.

One case series and two case reports show evidence that salvage of 7DS is possible with immune modulating therapy. Zhongwei et al. suggested that IV methylprednisolone treatment may delay occurrence and reduce mortality from 7DS. Matsuura et al. demonstrated that rituximab, plasma exchange and IV immunoglobulin was effective in salvaging a case of acute graft failure that was consistent with 7DS (20).

Universal recognition of what comprises 7DS is lacking and the establishment of accepted features may improve the recognition and reporting. The underlying pathological process that results in the clinical syndrome of 7DS could be a single entity, or several different disease states. A greater understanding of the cause may allow risk stratification and the implementation of preventative strategies. The proposed mechanisms of spontaneous early graft failure have evolved since the introduction of liver transplantation, especially in regard to the role of AMR, and these are subsequently described.

### Clinical Features of 7DS

**Figure 4:**
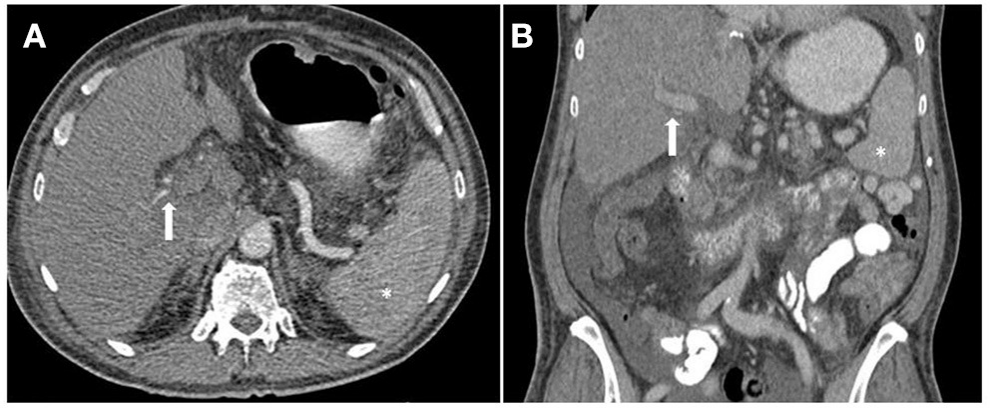
Computed tomography (CT) image of patient with Seventh-Day Syndrome. Axial **(A)** and coronal **(B)**. **(A)** The arterial phase axial image on the left demonstrates a patent right and left hepatic artery (arrow) and a relatively hypoenhancing liver in comparison to the spleen (*). **(B)** The coronal image in the portal venous phase demonstrates a patent portal vein.

There does not appear to be a single pathognomonic feature of this clinical syndrome. Based on all reported cases of 7DS, we suggest that in addition to acute graft failure the two following features must be present; (1) Initial graft function and (2) evidence of vascular patency on contrast enhanced CT. Features strongly supportive of 7DS include a rapid rise in ALT >1,000 IU/L after post-operative day 4, hypoenhancement of the liver on an arterial phase CT (Figure 4), and no clinical improvement with high dose corticosteroids. Alongside these, severe systemic instability occurs and lactic acidosis, severe vasoplegia, hypoglycaemia, often accompanied by fever, are other added clinical features. An element of reporting bias may be present with only severe cases of 7DS being described in the literature. A milder form of 7DS may exist in which high-dose intravenous immunosuppression with methylprednisolone delays the progress of the syndrome, such as described by Hwang et al. (6) and Zhongwei et al. (7). Recovery of graft function may coincide with increased immunosuppression and therefore the cause for the deterioration may instead be attributed to acute TCMR. As mentioned previously, the label of 7DS is best applied to the clinical presentation, rather than the underlying histological diagnosis.

### Pointers to the Pathogenesis

When interpreting the early literature, it must be remembered that rejection is often a mixed type, so early papers on TCMR likely also include an element of AMR. In addition to anti-HLA antibodies, non-HLA antibodies, including ABO antibodies, have also been implicated in the humoral response against the liver graft and these are rarely measured in clinical practice (25, 26). Hübscher et al. (3), in the early series from our institution fitting the 7DS definition, introduced the term massive haemorrhagic necrosis (3) to describe the pathology of the failed allograft. They noted an overlap with “acute rejection” (now TCMR) and chronic rejection (foam cell arteriopathy and/or ductopaenia), now considered to be due to antibody mediated rejection. Additionally, an arteritis with fibrinoid necrosis, a feature suggestive of antibody mediated rejection was also seen. One of these cases was an ABO incompatible transplant in the era prior to desensitization therapy. Starzl et al. described a similar pathology in the clinical scenario they termed “septic hepatic gangrene,” occurring between post-operative day 2 and 104 (median of day 25) and concluded this to be a consequence of rejection resulting in areas of necrotic parenchyma with secondary bacterial infection by gut bacteria (16). Once again, segmental areas of fibrinoid necrosis in hepatic arterial branches were seen. In both of these early series there were sequential biopsies taken throughout the process which showed the evolution from less severe necrosis, in zone 3 (around central veins) which increased over time to involve the midzone with a zone 2 and 3 pattern. In the failed allografts, a more extensive “geographic” necrosis predominated.

Demetris, part of the Starzl team, assessed the pathology of ABOI transplants which failed early post-transplant, as a result of a delayed hyperacute AMR, and found similar features of geographic haemorrhagic necrosis with diffuse intraorgan coagulation. Tissue bound donor-specific antibody (antiagglutins) within the graft were able to be eluted, confirming that antibody mediated rejection does occur in the liver allograft with similar histological features to 7DS.

In 1995, McCaughan et al. concluded that a syndrome of early graft failure, fitting the 7DS definition, represented antibody mediated rejection requiring early recognition with urgent retransplantation being the only effective therapy. The four cases reported by Burke et al., had variable TCMR preceding and/or occurring in conjunction with the development of a predominantly zone 3 hepatocyte necrosis with hemorrhage (19). They identified extensive endothelial injury to the hepatic veins associated with preceding high levels of IFN-γ and TNF-α followed by a rise in IL-6, at lower levels than that seen in steroid responsive TCMR. Interferon-γ and TNF are produced by macrophages and natural killer cells as part of AMR (27).

The early era pointed toward rejection as the cause, including delayed “hyperacute” antibody mediated rejection, however, presumably due to its rare occurrence and the delayed nature of AMR, it was overlooked as a potential cause of this early graft loss. In 2001, when the term 7DS was introduced for this clinical syndrome, specific pathways were assessed. These were based in animal studies of pathways producing hemorrhagic necrosis. Memon et al. proposed that an antibody against the *Fas* cell surface antigen was implicated in the process (1), based on a murine study that injecting an anti-fas receptor antibody caused panacinar hepatocyte necrosis and focal hemorrhage (28) *via* an apoptotic pathway which was universally fatal to the mice. A single study by Zhongwei et al. (7) assessed Fas staining in 7DS, finding it strongly expressed in periportal hepatocytes, but not in the areas of coagulative necrosis. This cell-death pathway has been demonstrated to be active during acute liver graft rejection (29).

Further clinical evidence that AMR contributes to this syndrome is a recent report of successful graft salvage in a case of 7DS, with therapies normally applied to refractory AMR, suggesting this process may be implicated in the syndrome (20). Previously described cases of acute graft failure, as a result of AMR, had some similar features to those described in our 7DS cases (30, 31). A role for AMR is supported by the finding of O'Leary et al. (32) where it was found that AMR was a contributor to previously unexplained early liver allograft loss. Contributing to the difficulty in diagnosing AMR in liver transplantation is the varied histological features and the fact it can affect different aspects of the graft vasculature. A recently described case of post-transplant sinusoidal obstruction syndrome attributed to AMR is a good example (33). These authors demonstrated venulitis of the central veins rather than the portal tracts, positive C4d staining of the sinusoids and positive DSAs (33). This patient's histological changes showed minimal improvement with steroid pulsing and defibrotide, however the initiation of plasmapheresis and IVIG therapy improved was effective.

Further research is required to understand the pathogenic mechanisms that initiate 7DS. However, based on the time of onset, histological and clinical features our opinion is an antibody mediated immune response against the graft is likely responsible.

### Summary

Recipients of both living and deceased donor grafts are at risk of 7DS. The typical pathology points toward rejection with intravascular thrombi as the cause in the majority of cases. It is characterized by a variable severity of TCMR, which often precedes the syndrome, however it does not respond to standard TCMR therapy of corticosteroids and calcineurin inhibitors. This therapy may modify the subsequent histopathological changes removing any co-existing TCMR component. Early biopsies may be subtle with focal centrilobular (zone 3) coagulative necrosis, with a minimal or absent haemorrhagic component. Over the ensuing days the extent of the necrosis increases, though largely maintaining a zonal distribution and involving zone 2 (midzonal) with increasing haemorhage into these areas. By the time of retransplantation (or post-mortem) it is predominantly zone 2 and 3 with several areas of geographic necrosis and prominent hemorrhage. Findings rarely seen in needle biopsies, and more readily identified in the failed allografts, are an arteritis with or without fibrinoid necrosis and intrahepatic thrombosis. Thrombi may occur in any of the portal and hepatic veins, sinusoids and intrahepatic arteries. Other features of AMR such as portal oedema, hemorrhage and microvasculitis have rarely been documented, but as these are subtle and AMR has only relatively recently been incorporated in the Banff criteria, this may easily have been overlooked. Other risk factors for intravascular thrombi may also play a role eg donor vascular disease or prothrombotic condition in the recipient. To delineate the underlying mechanisms conclusively, we recommend routine thorough histological analysis of explanted grafts or biopsies, which should include immunohistochemistry. The findings of these should then be reported in publications on the topic. The rare incidence, but clinical importance, of this syndrome necessitates international collaboration to corroborate individual institution findings and move toward greater understanding.

In conclusion, 7DS is a clinical syndrome of early graft failure, following a period of initial graft function, with patent vasculature on imaging. Following a comprehensive literature review and appraisal of our previous and current institutional patient series, we propose that the pathogenesis relates to intragraft microthrombi formation, most commonly as part of antibody mediated rejection. Confirmation of this hypothesis and a greater understanding of the mechanisms involved in 7DS requires routine assessment of sensitization pretransplant and monitoring of donor specific antibodies post-transplant in the sensitized patients. It also requires early recognition of the syndrome so that a biopsy can be performed before the patient is too unwell. It is imperative that all clinicians in the multidisciplinary team managing liver transplant recipients are aware of this rare syndrome and its implications, the requirement for prompt diagnosis and retransplantation. International multicentre trials on alternative immunosuppressive therapies are required if the role of AMR is confirmed.

## Data Availability Statement

The datasets presented in this article are not readily available because of institutional restriction—data protection. Requests to access the datasets should be directed to James.Halle-SMith@uhb.nhs.uk.

## Ethics Statement

Ethical review and approval was not required for the study on human participants in accordance with the local legislation and institutional requirements. Written informed consent for participation was not required for this study in accordance with the national legislation and the institutional requirements.

## Author Contributions

JH-S, LH, AH, HH, MP, and DN devised the research question and methodology, wrote, and reviewed the manuscript. JH-S, LH, and AH collected and analyzed the data. All authors contributed to the article and approved the submitted version.

## Conflict of Interest

The authors declare that the research was conducted in the absence of any commercial or financial relationships that could be construed as a potential conflict of interest.

## Publisher's Note

All claims expressed in this article are solely those of the authors and do not necessarily represent those of their affiliated organizations, or those of the publisher, the editors and the reviewers. Any product that may be evaluated in this article, or claim that may be made by its manufacturer, is not guaranteed or endorsed by the publisher.

## Abbreviations

7DS: seventh-day syndrome
ALT: alanine aminotransferase
AST: aspartate aminotransferase
BPAR: biopsy proven acute rejection
DBD: deceased donor after brain death
DCD: deceased donor after circulatory death
IRI: ischaemia-reperfusion injury
PNF: primary non-function
